# Discovery of New Inhibitors of Transforming Growth Factor-Beta Type 1 Receptor by Utilizing Docking and Structure-Activity Relationship Analysis

**DOI:** 10.3390/ijms20174090

**Published:** 2019-08-21

**Authors:** Jun-Hao Jiang, Ping Deng

**Affiliations:** College of Pharmacy, Chongqing Medical University, No. 1 Yixueyuan Road, Yuzhong District, Chongqing 400016, China

**Keywords:** TGF-beta, docking, structure-activity relationship, inhibitors, discovery studio, TGF-β, TβR1

## Abstract

The transforming growth factor-beta (TGF-β) plays an important role in pathological fibrosis and cancer transformation. Therefore, the inhibition of the TGF-β signaling pathway has therapeutic potential in the treatment of cancer. In this study, the binding modes between 47 molecules with a pyrrolotriazine-like backbone structure and transforming growth factor-beta type 1 receptor (TβR1) were simulated by molecular docking using Discovery Studio software, and their structure–activity relationships were analyzed. On the basis of the analysis of the binding modes of ligands in the active site and the structure–activity relationships, 29,254 new compounds were designed for virtual screening. According to the aforementioned analyses and Lipinski’s rule of five, five new compounds (CQMU1901–1905) with potential activity were screened through molecular docking. Among them, CQMU1905 is an attractive molecule composed of 5-fluorouracil (5-FU), 6-mercaptopurine (6-MP), and 5-azacytosine. Interestingly, 5-FU, 6-MP, and 5-azacytidine are often used as anti-metabolic agents in cancer treatment. Compared with existing compounds, CQMU1901–1905 can interact with target proteins more effectively and have good potential for modification, making them worthy of further study.

## 1. Introduction

Transforming growth factor-beta (TGF-β) is an important member of the TGF-β superfamily and plays an important role in pathological fibrosis and cancer [1]. In states of diabetic nephropathy, Crohn’s disease, myocardial fibrosis, and idiopathic pulmonary fibrosis (IPF), an increase in TGF-β is a powerful factor associated with disease progression [2]. TGFβ-signaling mediates protumorigenic changes in the tumor microenvironment (TME) and promotes epithelial-to-mesenchymal transition (EMT), both of which aid in tumor progression and invasiveness [3,4]. In addition, the TGFβ-signaling pathway has been reported to play a crucial role in the later stages of tumorigenesis via raising immunosuppressive Treg cells [5].

TGFβ signaling exerts physiological effects through two transmembrane serine/threonine kinase receptors, transforming growth factor-beta type 1 receptor (TβR1) and transforming growth factor-beta type 2 receptor (TβR2). When TGF-β binds to TβR2, the binding compound is recognized by TβR1 and forms a tetramer complex consisting of two TβR1 and two TβR2. The formation of the complex allows TβR2 to cross phosphorylate TβR1, which results in its activation and subsequent recruitment and phosphorylation of the mothers against decapentaplegic (SMAD) proteins. The phosphorylation of SMAD leads to the dimerization of SMAD, the translocation to the nucleus, followed by gene transcription, and then it gives rise to a series of physiological effects [6].

The TGFβ-signaling pathway plays an important role in the development of pathological fibrosis and cancer, and TβR1 is the key site of the TGFβ-signaling pathway. Thus, as a potential drug target, TβR1 has attracted wide attention [1]. Galunisertib (LY2157299), a new TβR1 inhibitor developed by Eli Lilly and Company (LLY), has been used in clinical trials as a therapeutic drug for myelodysplastic syndrome and primary hepatocellular carcinoma. In addition, the combination of Galunisertib and Nivolmab, a new inhibitor of programmed cell death-1 (PD-1) from Merck Sharp and Dohme (MSD), for the treatment of bone marrow hyperplasia and hepatocellular carcinoma, has entered a phase III multicenter clinical trial [7]. EW-7197, a TβR1 inhibitor developed by Ewha Womans University with potential clinical therapeutic value for melanoma, breast cancer, and liver fibrosis, is undergoing clinical trials. The newly developed TβR1 inhibitor with a pyrrolotriazine-like backbone structure from Bristol-Myers Squibb (BMS) has high selectivity and activity, potential clinical application prospects, and economic value. BMS has submitted two patent applications for invention in China, No. 201680055202.3 and No. 201680049890.2, which have entered substantive examination [8,9]. In recent years, some other new TβR1 inhibitors have been reported, such as SB-431542, GW-788388, R-268712. From this point of view, the design of small molecule inhibitors of TβR1 with high efficiency and low toxicity has become a research hotspot with broad market prospects.

**Figure 1:**
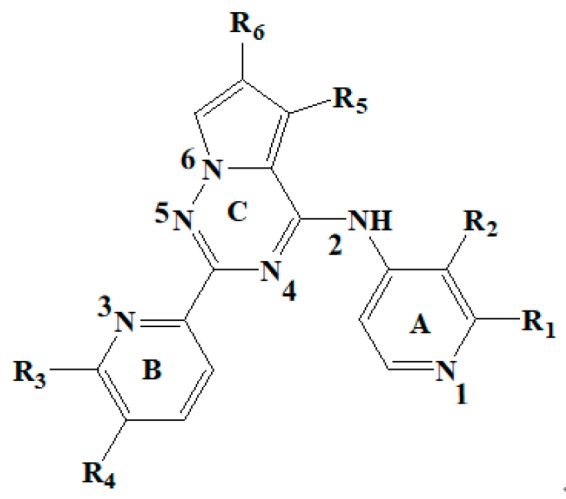
Structure of compounds with a pyrrolotriazine-like backbone as TGF-β type 1 receptor (TβR1) inhibitors; the three rings are labeled A, B, and C, and the nitrogen atoms are specified as 1, 2, 3, 4, 5, and 6.

With the rapid development of computer software and hardware technology, molecular docking research has been widely used in innovative drug design [10,11]. There is a previous study of ours related to the present work [12] in which two new skeleton structures were found by searching databases, and, subsequently, three compounds (YXY01–03) with certain activity and high safety were designed. However, in this study, we try to analyze the interaction modes and structure–activity relationships of the highly active compounds in order to aid the design of new drugs. Two virtual screenings are different means, but they complement each other to find more new inhibitors. In this study, the newly developed TβR1 inhibitor with a pyrrolotriazine-like backbone structure (Figure 1 and Table S1) has high selectivity and activity, potential clinical application prospects, and economic value. Among them, Compound 1, with an IC_50_ (half maximal inhibitory concentration) value of 0.25 nM, is the most active TβR1 inhibitor reported at present. Forty-seven compounds with a pyrrolotriazine-like backbone structure were analyzed with docking experiments using Discovery Studio (DS) software in terms of their interaction with the receptor, using its crystal structure. The structure-activity relationships were then analyzed for all compounds to identify the optimal candidates for further analysis. The atomic-resolution details of drug-receptor interactions were used to propose variations in the chemical structure of the compounds to optimize the interaction, including critical hydrophobic and H-bonding contacts. The structure-activity data allowed us to propose a large number of new compounds which were then narrowed down based on certain pharmacological criteria, and eventually the highly promising compounds were identified for real drug screening and clinical studies. This study can provide theoretical guidance for the design of subsequent active inhibitors of TβR1.

## 2. Results and Discussion

### 2.1. Docking Results and Binding Mode

**Figure 2:**
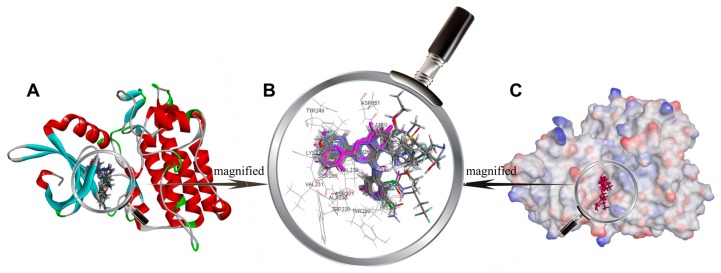
(**A**) Docked ligands in the binding pocket of TGF-β type 1 receptor (TβR1) displayed with solid ribbon, colored according to secondary structure, helices in red, beta sheets in cyan, turns in green, and coils in white. (**B**) binding of ligands in the active site—the purple one is a crystal structure pose of BMS22 (TβR1 inhibitor from Bristol-Myers Squibb, structure of which is detailed in Table S1) from the Protein Data Bank (PDB) ID: 6B8Y; (**C**) receptor surface colored by the interpolated atomic charge—blue represents a positive value and red represents a negative value.

In order to verify the reliability of the CDOCKER docking results, BMS22 and 47 inhibitors were docked to the active sites of TβR1 under the same docking conditions. The docking results show that the conformation of BMS22 obtained by CDOCKER docking was highly consistent with that of crystal, and the minimum root mean square deviation (RMSD) of heavy atoms was 0.2879, which indicates that the docking method is feasible and the docking results are credible. The active conformations of 47 molecules were obtained by the screening criteria of a higher docking score and similar binding mode with BMS22 in the crystal structure. Their conformations of ligands in the binding pocket of TβR1 are shown in Figure 2

**Figure 3:**
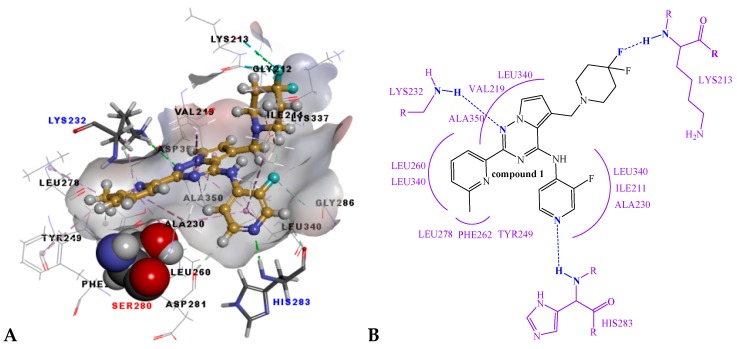
(**A**) The interpolated charge receptor surface (amino acid residue SER280 in the Corey-Pauling-Koltun model, amino acid residue LYS232 and HIS283 in the stick model, other amino acid residues in the line model and Compound 1 in the ball and stick model; the receptor surface colored by the interpolated atomic charge—blue represents a positive value and red represents a negative value); (**B**) the ligand–receptor interactions of Compound 1 shown as a 2D diagram—blue dotted lines represent hydrogen bindings and purple curved lines represent the frontiers of amino acid residues binding to Compound 1.

Inhibitors usually bind to target proteins by electrostatic, hydrogen-bonding and hydrophobic interactions with amino acid residues around the active site. The receptor surface schematic diagram is shown in Figure 2C and Figure 3A. As shown in Figure 3A, residue SER280 protrudes into the cavity of the receptor site and forms a Y-type binding pocket with residue SER280 as a convex point. Therefore, most TβR1 inhibitors are Y-shaped, which can coincide with the Y-type binding pocket in the receptor. Though there is no interaction between residue SER280 and the ligands in terms of crystal structure and the binding mode obtained by molecular docking, residue SER280 is an important structural feature of the binding pocket.

The binding mode of Compound 1 with the highest activity is shown in Figure 3. As illustrated in Figure 3A, amino acid residues such as ILE211, ALA230, and LEU340 form a cavity, and Ring A is inserted into it to form a stable spatial structure through hydrophobic interaction. Amino acid residues such as LYS232, LEU260 and LEU340 form a hydrophobic cavity, and Ring B extends into the cavity to form another stable spatial structure through hydrophobic interaction. In addition, a stable hydrophobic interaction was formed between Ring C and residues VAL219, LEU340, and ALA350. Aside from the hydrophobic effects, two important hydrogen bonds were formed between the ligand and the certain amino acid residues. The N1 atom on Ring A and residue HIS283 form an N···H-N hydrogen bond, while the N5 atom on Ring C and LYS232 form another N···H-N hydrogen bond.

On the basis of the above analysis, there are two characteristics of the binding mode between ligands and TβR1: (1) The hydrophobic interactions between three aromatic ring regions and amino acid residues are the key factors in the binding process; (2) the docking conformations of most active molecules indicate that the hydrogen bonds formed between the N1 and N5 atoms and the amino acid residues may be the crucial sites for pharmacological activity. On the premise of retaining the hydrogen bonds between the N1 and N5 atoms and the amino acid residues, new inhibitors may be found by modifying the molecular skeleton.

### 2.2. Structure—Activity Relationships

**Figure 4:**
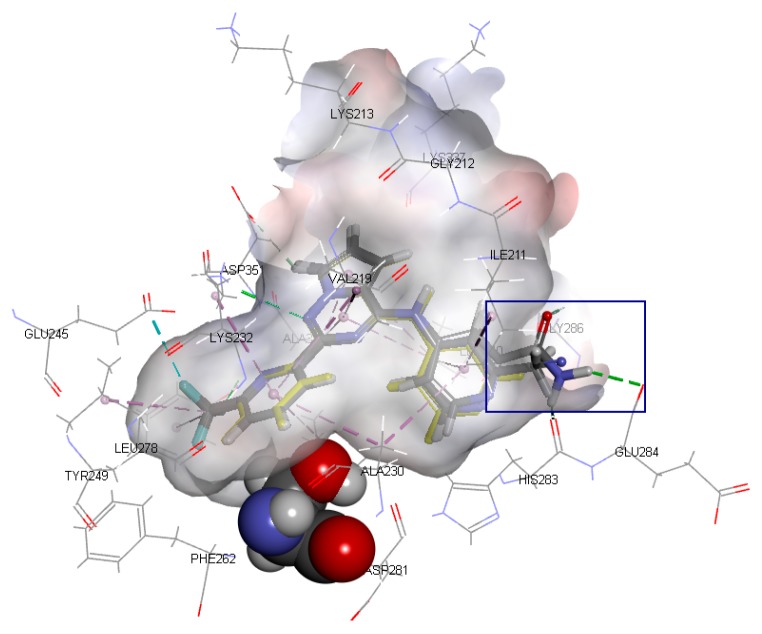
The binding mode of Compounds 2, 3, 4, 5, and 6 at the active site (amino acid residue SER280 in the Corey-Pauling-Koltun model, other amino acid residues in the line model and ligands in the stick model, among which Compound 2 is expressed in yellow; the receptor surface colored by the interpolated atomic charge—blue represents a positive value and red represents a negative value; the R1-position on ring A highlighted in a blue rectangle).

In order to investigate the substitution effect in the R1-position of Ring A, the docking conformation, molecular structure, and activity of the molecules with different substituents were compared and analyzed (Figure 4 and Table 1). As listed in Table 1, the activity decreased significantly when the H atom in the R1-position of Compound 2 was substituted by other substituents. The introduction of -CH_3_, -CONH_2_, and -CN groups resulted in steric hindrance, which increased the space distance between the N1 atom and residue HIS283, and may have led to the disappearance or weakening of the N···H-N hydrogen bond, resulting in a decrease in activity. For example, when the hydrogen bonds between the N1 atoms and residues HIS283 in Compounds 3 and 4 disappeared, the pIC_50_ (logIC_50_) values reduce by 2.1474 and 2.2773, respectively, these values both being lower than Compound 22. The hydrogen bond length between the N1 atom in Compound 5 and residue HIS283 increased by 0.11 Å, then the hydrogen bond interactions were weakened, and the pIC_50_ value decreased by 1.8842. The position of R1 was close to the electronegativity region of the receptor surface formed by amino acid residue HIS283, and the electronegativity groups in the R1-position, such as -F (Compound 6), may have caused electrostatic repulsion and decreased the activity of the receptor surface.

In conclusion, the steric hindrance or electrostatic repulsion caused by the substituent in the R1-position may have significantly reduced the activity, which was a disadvantageous site for the structural modification of compounds.

**Figure 5:**
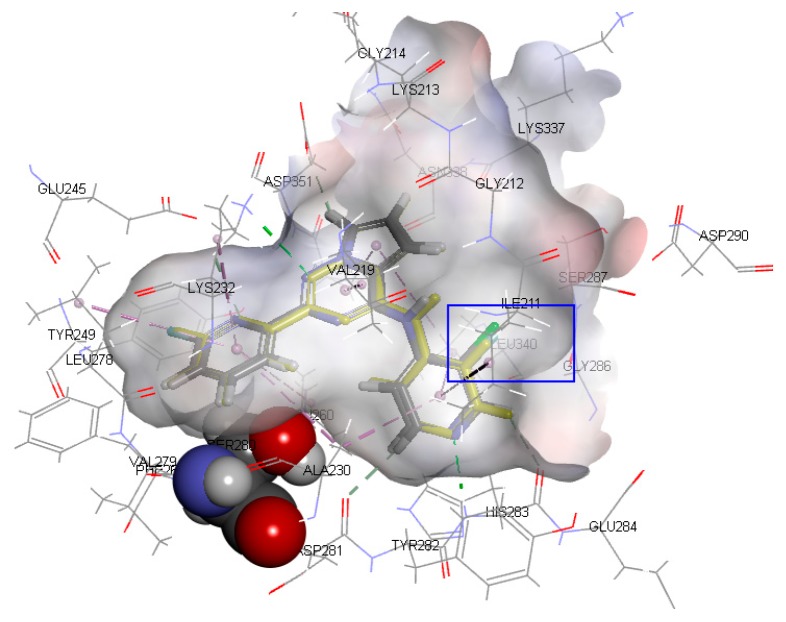
The binding modes of Compounds 7, 8, and 9 at the active site (amino acid residue SER280 in the Corey-Pauling-Koltun model, other amino acid residues in the line model and ligands in the stick model, among which Compound 7 is expressed in yellow; the receptor surface colored by the interpolated atomic charge—blue represents a positive value and red represents a negative value; the R2-position on ring A highlighted in a blue rectangle).

If there are small hydrophobic groups such as -Cl and -F in the R2-position of Ring A, the activity of the compound may significantly increase (data listed in Table 2). The pIC50 value increased by 1.5550 or 1.2485 when -H in the R2-position of Compound 7 was replaced by -Cl (Compound 8) or -F (Compound 9). In addition, the values of pIC_50_ increased by 0.1930 and 0.2218, respectively, after substituting -H in the R2-position of Compound 10 for -Cl (Compound 11) and -F (Compound 12). The value of pIC_50_ increased by 0.2076 after replacing -H in the R2-position of Compound 13 with -F (Compound 14). The analysis of the binding mode (Figure 5) illustrated that the halogen atoms in the R2-position can enhance the hydrophobic interaction with amino acid residues, which is conducive to ligand–receptor binding. Furthermore, the steric hindrance caused by the substituent in the R2-position could decrease the activity. For example, Compounds 15, 16, and 17 showed lower activity.

Considering all of these points, the conclusion can be drawn that the existence of small electronegative groups in the R2-position of cyclic A is beneficial in terms of increasing the activity, and the introduction of other groups should be considered in combination with steric hindrance and other factors.

As shown in Figure 3, Ring B lied deep in the cavity of the receptor and was surrounded by hydrophobic amino acids. The hydrophobic group on Ring B was beneficial to increase the activity of the compounds; nevertheless, the activity decreased as a result of the polar group or steric hindrance caused by the substituent exists (Table 3). For example, the pIC_50_ value of Compound 9 increased by 0.2785 compared to Compound 12.

On the basis of the characteristics of the cavity structure, the steric hindrance caused by the substituent groups in the R3-position of Ring B was a disadvantage factor; meanwhile, the small hydrophobic groups in the R3-position may have increased hydrophobic interaction and enhanced activity. The R4-position was close to the SER280 group, and the presence of substituents could form steric hindrance, which was a disadvantage factor for activity.

The R5-position of Ring C was located at the outer side of the active site cavity; thus, different substituents could be introduced to modify the structure. Different substituents may interact with different amino acid residues, but there was no obvious regularity between activity changes and various substituents.

The R6-position of Ring C was very close to the LYS337 residue, and the existence of substituents tended to form steric hindrance, reducing activity. For example, compared with Compound 13, the activity of Compound 17 with -COOC_2_H_5_ in the R6-position decreased significantly, and compared with Compound 23, the activity of Compound 20 with -NS_2_O_4_CH_3_ in the R6-position also decreased distinctly. Therefore, it was disadvantageous to add substituent groups in the R6-position in order to increase the activity of the compounds.

To summarize, the R1-, R4-, and R6-positions were close to amino acids HIS283, SER280, and LYS337 of the receptor, respectively, and the steric hindrance caused by the substituent group could significantly decrease the activity, which was an unfavorable site for structural modification of the compounds. The hydrophobic groups in the R2- or R3-position could increase hydrophobicity and enhance activity. However, the R5-position was near the outer side of the active site cavity, and so different substituent groups can be introduced in this position, modifying the structure to increase the activity or improve the physical and chemical properties of the inhibitors.

### 2.3. Design and Screening of New Inhibitors

**Figure 6:**
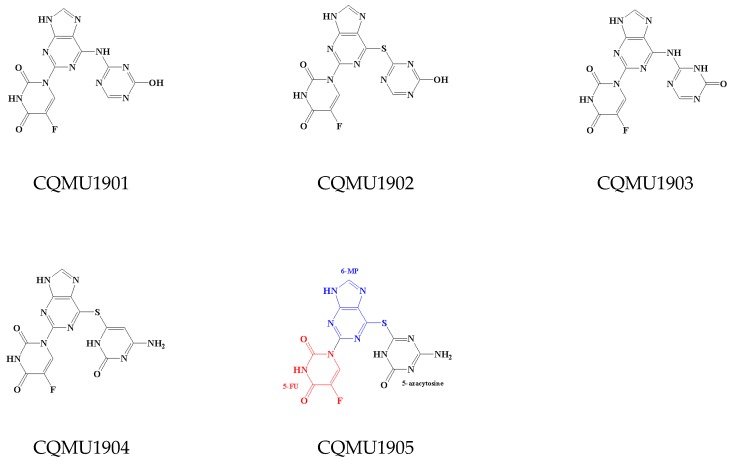
The structures of novel inhibitors CQMU1901–1905 (the 6-MP fragment in blue, the 5-FU fragment in red and the 5-azacytosine fragment in black).

On the basis of the analysis of the binding modes of ligands in the active site and structure-activity relationships, combined with the characteristics of pharmacophore [12], 29,254 new compounds were designed for virtual screening using the reaction-based in situ enumeration method and scaffold hopping methods with DS software and experience-based manual design. To make the retrieved compounds more drug-like, the selected hit compounds were required to meet Lipinski’s rule of five. After that, a set of 9254 compounds were selected from the 29,254 compounds by Lipinski’s rule of five for the subsequent CDOCKER docking study. All compounds were docked into the active site of 6B8Y using CDOCKER docking. Novel inhibitors with a lower CDOCKER energy and a similar action mechanism were selected for the next study. Finally, five novel inhibitors (CQMU1901–1905) with potential activity were screened. Their structures are illustrated in Figure 6, and relevant data are listed in Table 4.

The novel inhibitors have the following characteristics: (1) Structural modification with purine as the mother nucleus, which is obviously different from the existing compound structure, can effectively break through patent protection; (2) like the existing molecules, the skeleton structures of the novel inhibitors have three aromatic rings which can form hydrophobic interaction with three regions of the receptor; (3) in addition to the hydrogen bonds between ligands and residues LYS232 and HIS283, the novel inhibitors can also form new hydrogen bonds with other amino acid residues in the active site, which is conducive to enhance the interaction between ligand and receptor.

**Figure 7:**
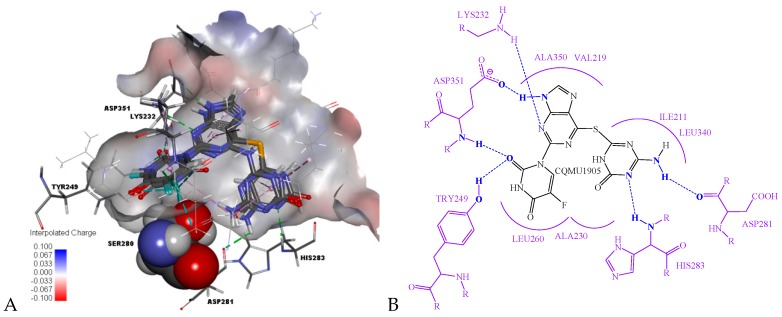
(**A**) The binding modes of Compounds CQMU1901–1905 in the active site (amino acid residue SER280 in the Corey-Pauling-Koltun model, amino acid residue LYS232, TYR249, AS281, HIS283, ASP351 and ligands in the stick model, and other amino acid residues in the line model; the receptor surface colored by the interpolated atomic charge—blue represents a positive value and red represents a negative value); (**B**) the receptor-ligand interactions of Compound CQMU1905 shown as a 2D diagram—blue dotted lines represent hydrogen bindings and purple curved lines represent the frontiers of amino acid residues binding to Compound CQMU1905.

Among them, CQMU1905 is a very interesting molecule for the following reasons: (1) It is composed of 5-fluorouracil (5-FU), 6-mercaptopurine (6-MP), and 5-azacytosine, and, as we all know, 5-FU, 6-MP, and 5-azacytidine are often used as anti-metabolic agents in cancer treatment; (2) as illustrated in Figure 7B, compared with Ring A of Compound 1, the azacytosine ring of CQMU1905 can form N–H···O hydrogen bonds between -NH2 in azacytosine ring and residue LYS281, as well as hydrogen bonds between the N atom on the azacytosine ring and residue HIS283; compared with Ring B of Compound 1, two hydrogen bonds were formed between carbonyl group in 5-FU of CQMU1905 and residue ASP351 and TYR249. Aside from this, there are halogen bonds between the F atom and residue ALA230 and LEU278, and compared with Ring C, the 6-MP of CQMU1905 not only forms hydrogen bonds with residue LYS232, it also forms hydrogen bonds with residue ASP351; (3) the -NH_2_ group of azacytosine is located at an excellent site to modify the molecular, chemical, and metabolic properties because the -NH_2_ group can form acylamides easily with fatty acids—thus, the properties of molecules such as LogP can be improved, the deamination effect can be reduced, and the reaction time can be increased.

## 3. Materials and Methods

### 3.1. Molecular Docking

The inhibitors with a pyrrolotriazine-like backbone structure of TβR1 (Figure 1) from China Patent 201680055202.3, which are the latest TβR1 inhibitors developed by BMS, were selected in this study. The physiological activity data of these compounds were derived from the same test group and with the same test conditions [9]. The starting conformations were constructed using the Gauss View 5.0 program. With the density functional theory (DFT) method at the B3LYP/6-31 + G(d) level, the molecules were optimized using the Gaussian 09 program. To simulate real conditions, the solvent effects of H_2_O were studied using the polarized continuum model (PCM).

The first crystal structure of the TβR1 protein was reported in 1999 (Protein Data Bank (PDB) ID: 1B6C) [13]. Up until now, the x-ray crystal structure of TβR1 with a number of small molecular inhibitors has been reported in the PDB database (http://www.rcsb.org), including 1VJY, 1PY5, 2WOT, 2X7O, 3KCF, 3FAA, 3TZM, 3GXL, 5QIK, 5QIN, and 6B8Y. The inhibitor (BMS22) in 6B8Y was highly similar in structure to the inhibitors studied in this paper. The protein receptor from 6B8Y was used for docking all compounds after undergoing the protein preparation processes, such as supplementing amino acid residues and adding hydrogen atoms. The dock ligands (CDOCKER) protocol, which is a grid-based molecular docking method that employs CHARMm [14] in DS software (https://www.3dsbiovia.com/), was used in this study. The receptor binding sites were determined from the PDB site records based on the ligand molecule BMS22 in the known location. To validate the docking reliability, BMS22 was first re-docked to the binding site. Consequently, all compounds were docked into the same active site, and then twenty conformations of each compound were obtained through CDOCKER.

### 3.2. Structure-Activity Relationship

A set of 47 molecular conformations with lower CDOCKER energy and a reasonable binding mode were selected for the next study. The IC_50_ values of Compounds 1–47 were derived from the China Patent literature 201680055202.3 [9]. In order to analyze the structure-activity relationship (SAR), the IC_50_ values of the compounds were converted into pIC_50_ (−logIC_50_). After that, the pIC_50_ values were distributed between −3.2304 and 0.6021. Pairs of molecules, differing in a single localized structural change [15] but provoking an activity change (ΔpIC_50_), were used in the subsequently SAR studies.

### 3.3. Compound Libraries

Compound libraries used for virtual screening in this research included 29,254 new compounds obtained in three ways: (1) The reaction-based ligand enumeration within the 6B8Y active site for lead optimization used the grow scaffold protocol. Beginning with Compound 1 positioned in the binding site of 6B8Y, the N2, R2, and R5 were selected as the positions to act as reaction vectors for the enumeration; (2) novel compounds with different scaffolds were obtained by replacing Rings A, B, C, and R5 of Compound 1 while maintaining a favorable binding, such as hydrogen bonds and hydrophobic interactions between 6B8Y and Compound 1; (3) according to the results of the structure–activity relationships and receptor-ligand binding modes, the structures of Compound 1 and BMS22 were manually modified to produce novel inhibitors based on experience.

To make the retrieved compounds more drug-like, the selected compounds were required to meet Lipinski’s rule of five. Thereafter, a set of 9254 compounds was selected from the 29,254 compounds for the subsequent CDOCKER docking study. All compounds were docked into the active site of 6B8Y using the CDOCKER module. Novel inhibitors with a lower CDOCKER energy and a similar action mechanism were selected for the next study. Finally, the five candidates CQMU1901~1905 with potential activity were obtained with completely different structures from that of present active compounds.

## 4. Conclusions

In this study, the binding modes of 47 inhibitors with a pyrrolotriazine-like backbone structure and TβR1 were simulated by molecular docking using DS software, and their structure-activity relationships were analyzed. On the basis of the analysis of the binding model of the ligands in the active site and the structure-activity relationship, 29,254 new compounds were designed for virtual screening. Five new compounds (CQMU1901–1905) with potential activity were finally obtained by virtual screening through molecular docking combined with Lipinski’s rule of five. Among them, CQMU1905 is a very interesting molecule composed of 5-FU, 6-MP, and 5-azacytosine. Compared with existing inhibitors, CQMU1901–1905 can interact with target proteins more effectively and have good potential for modification, which makes them worthy of further study. The potentially active compounds could be synthesized and evaluated.

## Figures and Tables

**Table 1:** The structure-activity relationships in the R1-positon on Ring A.

NO.	MS	pIC_50_	ΔpIC_50_	NO.	MS	pIC_50_	ΔpIC_50_
2	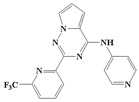	−0.6021	/	5	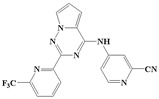	−2.5563	–1.8842
3	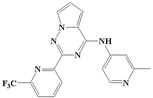	−2.8195	–2.1474	6	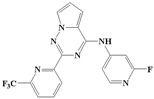	−2.3979	–1.7258
4	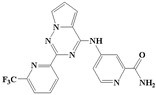	−2.9494	–2.2773				

NO.: Number of compounds; MS: Molecular structure; pIC_50_: −logIC_50_ (IC_50_: Half maximal inhibitory concentration, nM); ΔpIC_50_: The difference value of pIC_50_ between Compounds 3, 4, 5, 6, and 2, respectively.

**Table 2:** The structure-activity relationships in the R2-positon on Ring A.

NO.	MS	pIC_50_	NO.	MS	pIC_50_
7	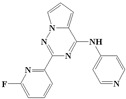	−1.1461	13	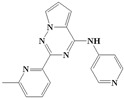	−0.1761
8	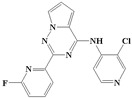	0.4089	14	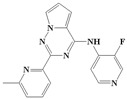	0.0315
9	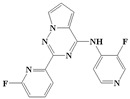	0.1024	15	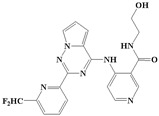	−0.5911
10	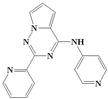	−0.3979	16	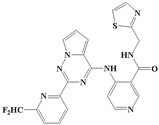	−1.6435
11	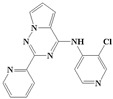	−0.2041	17	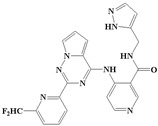	−1.6232
12	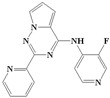	−0.1761			

NO.: Number of compounds; MS: Molecular structure; pIC_50_: −logIC_50_ (IC_50_: Half maximal inhibitory concentration, nM).

**Table 3:** The structure-activity relationships on Ring B.

NO.	MS	pIC_50_	ΔpIC_50_	NO.	MS	pIC_50_	ΔpIC_50_
12	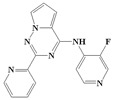	−0.1761	/	20	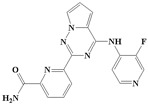	−1.8129	–1.6368
9	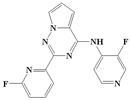	0.1024	0.2785	21	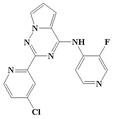	−2.1761	–2.0000
18	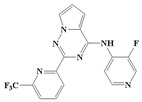	−0.5682	–0.3921	22	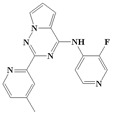	−3.2304	–3.0543
19	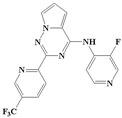	−1.8451	–1.6690				

NO.: Number of compounds; MS: Molecular structure; pIC50: −logIC50 (IC_50_: Half maximal inhibitory concentration, nM); ΔpIC50: The difference value of pIC50 between Compounds 9, 18, 19, 20, 21, 22, and 12, respectively.

**Table 4:** Parameters of Lipinski’s rule of five and CDDOCK energy of compounds CQMU1901–1905.

Name	MW	ALogP	ROTB	HBA	HBD	CDDOCK energy
CQMU1901	358	0.142	3	10	2	−52.7057
CQMU1902	375	0.710	3	10	1	−59.5115
CQMU1903	358	−0.791	3	9	1	−40.7534
CQMU1904	389	−0.311	3	9	2	−38.3008
CQMU1905	390	0.283	3	10	2	−48.0729

MW: Molecular weight; ALogP: Log of the octanol-water partition coefficient using Ghose and Crippen’s method; ROTB: Rotatable bond; HBA: Hydrogen bond acceptor; HBD: Hydrogen bond donor; CDDOCK energy: The score calculated by DS software—a more negative score is favorable for binding. The CDDOCK energy of BMS22 and Compound 1 are −32.0942 and −46.6792, respectively.

## Supplementary Materials

Supplementary materials can be found at https://www.mdpi.com/1422-0067/20/17/4090/s1.
