# Supplementary material for: Discovery of New Inhibitors of Transforming Growth Factor-Beta Type 1 Receptor by Utilizing Docking and Structure-Activity Relationship Analysis
Source: Int J Mol Sci. 2019 Aug 21;20(17):4090. doi: 10.3390/ijms20174090 (PMC6747504; doi:10.3390/ijms20174090)
Supplement: Supplementary file 1 [file ijms-20-04090-s001.pdf]

Supplementary

Table S1. The structure and activity of compound 1–47. NO., number of compounds; MS, molecular structure; pIC_{50}, $-logIC_{50}$; IC_{50}, half maximal inhibitory concentration, nM.

NO.	MS	pIC_{50}	IC_{50}* (nM)	NO.	MS	pIC_{50}	IC_{50}(nM)
1	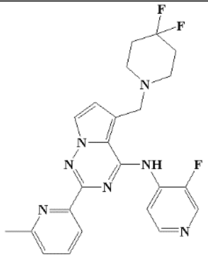	0.6021	0.25	25	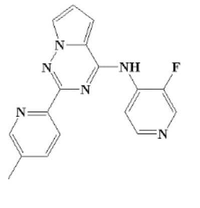	0.1024	0.79
2	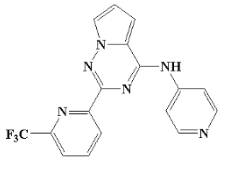	−0.6021	4.00	26	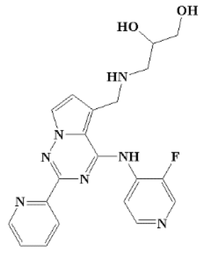	0.0757	0.84
3	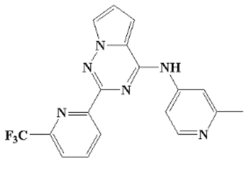	−2.8195	660.00	27	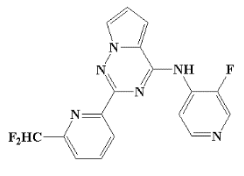	0.0362	0.92
4	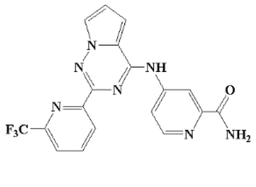	−2.9494	890.00	28	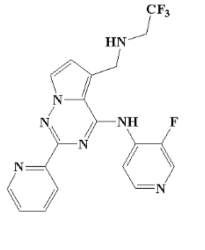	−0.0414	1.10
5	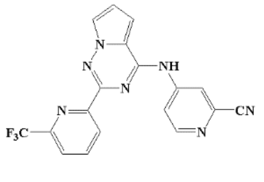	−2.5563	360.00	29	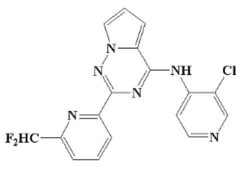	−0.0414	1.10

6	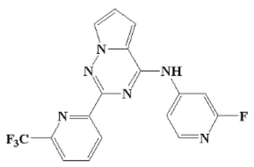	−2.3979	250.00	30	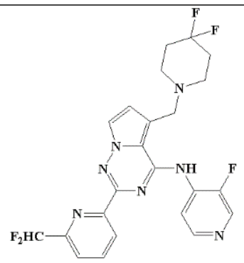	−0.0414	1.10
7	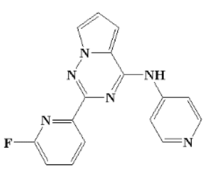	−1.1461	14.00	31	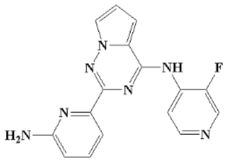	−0.2041	1.60
8	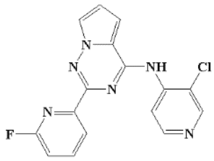	0.4089	0.39	32	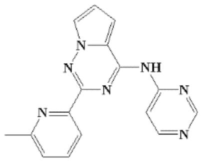	−0.3424	2.20
9	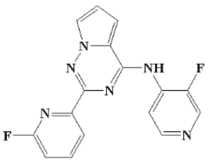	0.1024	0.79	33	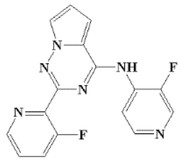	−0.5051	3.20
10	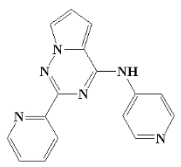	−0.3979	2.50	34	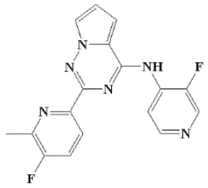	−0.7853	6.10
11	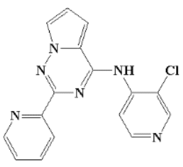	−0.2041	1.60	35	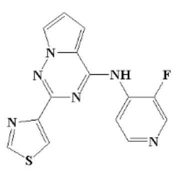	−0.9138	8.20
12	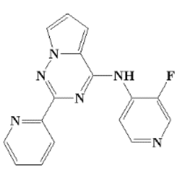	−0.1761	1.50	36	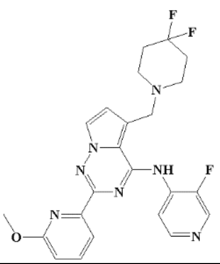	−0.9542	9.00

13	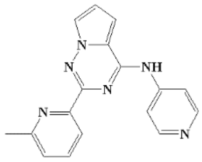	−0.1761	1.50	37	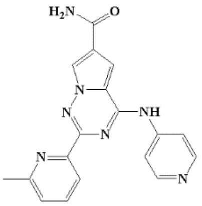	−1.0000	10.00
14	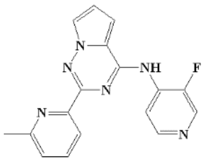	0.0315	0.93	38	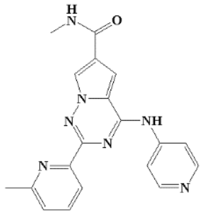	−1.0414	11.00
15	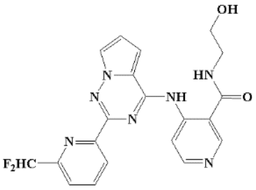	−0.5911	3.90	39	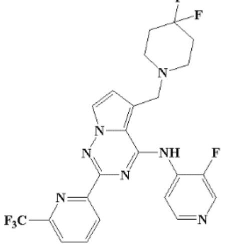	−1.1139	13.00
16	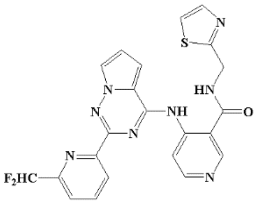	−1.6435	44.00	40	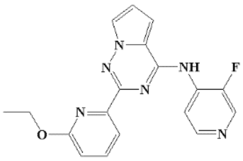	−1.1761	15.00
17	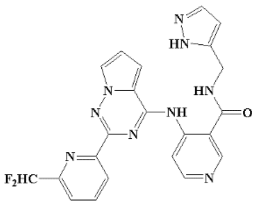	−1.6232	42.00	41	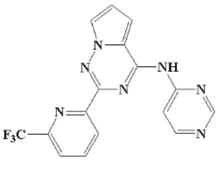	−1.4472	28.00
18	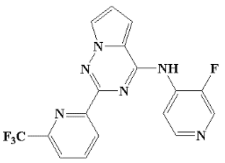	−0.5682	3.70	42	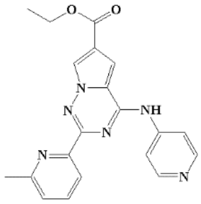	−1.5185	33.00
19	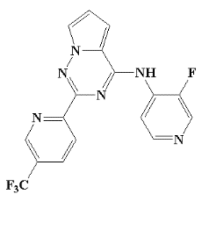	−1.8451	70.00	43	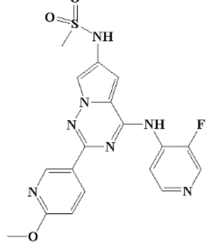	−1.7482	56.00

20	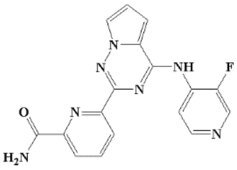	−1.8129	65.00	44	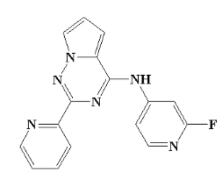	−1.8388	69.00
21	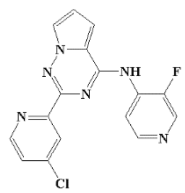	−2.1761	150.00	45	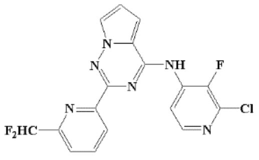	−1.9445	88.00
22	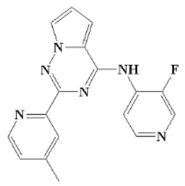	−3.2304	1700.00	46	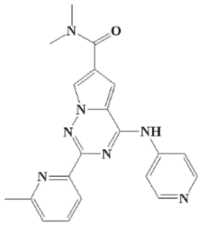	−2.1139	130.00
23	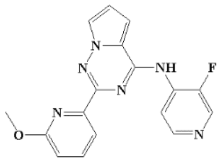	−0.6021	4.00	47	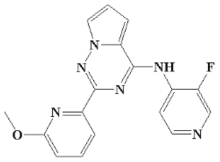	−2.7243	530.00
24	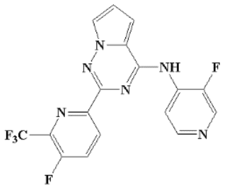	0.3372	0.46	BMS 22	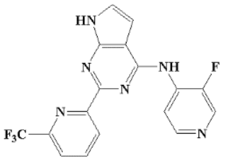	0.2596	0.55

The IC_{50} values of compound 1~47 are derived from the China patent 201680055202.3. In order to analyze the structure–activity relationship (SAR), the IC_{50} values of the compounds were converted into pIC_{50} ($-logIC_{50}$).
